# Snake Venom Metalloproteinases from Puff Adder and Saw-Scaled Viper Venoms Cause Cytotoxic Effects in Human Keratinocytes

**DOI:** 10.3390/toxins17070328

**Published:** 2025-06-28

**Authors:** Keirah E. Bartlett, Adam Westhorpe, Mark C. Wilkinson, Nicholas R. Casewell

**Affiliations:** Centre for Snakebite Research & Interventions, Department of Tropical Disease Biology, Liverpool School of Tropical Medicine, Liverpool L3 5QA, UK; keirah.bartlett@lstmed.ac.uk (K.E.B.); adam.westhorpe@lstmed.ac.uk (A.W.); mark.wilkinson@lstmed.ac.uk (M.C.W.)

**Keywords:** toxins, cytotoxicity, metalloproteinases, snakebite envenoming, neglected tropical diseases

## Abstract

Snakebite envenoming is a neglected tropical disease that causes substantial mortality and morbidity globally. The puff adder (*Bitis arietans*) and saw-scaled viper (*Echis romani*) have cytotoxic venoms that cause permanent injury via dermonecrosis around the bite site. Identifying the cytotoxic toxins within these venoms will allow for the development of targeted treatments to prevent snakebite morbidity. In this study, venoms from both species were fractionated using gel filtration chromatography, and a combination of cytotoxicity approaches, SDS-PAGE gel electrophoresis, and enzymatic assays were applied to identify the venom cytotoxins in the resulting fractions. Our results indicate that snake venom metalloproteinase (SVMP) toxins are responsible for causing cytotoxic effects across both venoms. The PI subclass of SVMPs is likely the main driver of cytotoxicity following envenoming by *B. arietans*, while the structurally distinct PIII subclass of SVMPs is mostly responsible for conveying this effect in *E. romani* venom. Identifying distinct SVMPs as cytotoxicity-causing toxins in these two African viper venoms will facilitate the future design and development of novel therapeutics targeting these medically important venoms, which in turn could help to mitigate the severe life- and limb-threatening consequences of tropical snakebites.

## 1. Introduction

Snakebite envenoming is a neglected tropical disease (NTD) with a high mortality and morbidity burden in rural regions of Sub-Saharan Africa, South and Southeast Asia, and Latin America [1]. Causing up to 138,000 deaths and 400,000 disabilities annually, snakebite envenoming has been designated as a World Health Organization (WHO) priority NTD since 2017, and an ambitious target has been set, aiming to halve the number of deaths and disabilities attributed to snakebite by 2030 [1,2]. Currently, the only specific therapeutic for snakebite is antivenom, and whilst invaluable in saving lives, this treatment often carries with it a range of affordability, efficacy, and safety issues [1,3]. Consequently, several new therapeutics are under investigation, such as monoclonal antibodies and small molecule inhibitors [4,5,6,7], for potently inhibiting key toxins within medically important venoms. However, it is critical that we first understand which venom components are of greatest pathogenic importance in medically important venoms to guide the discovery and development of more targeted and efficacious treatments [8].

While some venoms cause life-threatening systemic effects following snakebite, such as haemorrhage or respiratory paralysis, many patients experience severe local tissue damage in the region surrounding the bite, which often results in permanent sequelae, such as limb or digit impairment, and/or the requirement for surgical interventions, such as debridement of necrotic tissue [9,10]. In Africa alone, as many as 14,600 snakebite patients may require limb amputation in response to snakebite-induced local tissue damage each year [2,11]. In Africa, localised tissue necrosis following snakebite can largely be attributed to venoms from a range of spitting cobras (family Elapidae) and vipers (family Viperidae) [12]. The vipers primarily responsible for causing morbidity following envenoming are the puff adder (*Bitis arietans*) and saw-scaled vipers (e.g., *Echis ocellatus*, *E. romani*, *E. pyramidum* and others) [12,13]. In West Africa, *E. romani* (recently reclassified and previously known as *E. ocellatus* [14,15]) is one of the main biting species, and in Nigeria alone, it contributes to approximately 90% of severe envenomings [16]. Collectively, *Bitis arietans* are believed to cause a substantial number of severe envenomings in Eastern, Central, and Southern Africa [12]. Both of these venoms are characterised by causing extensive local tissue damage as the result of venom cytotoxicity, in addition to inducing systemic haemostatic disturbances, such as haemorrhage, coagulopathy, and hypotension [13,17].

Like many snake venoms, those of *E. romani* and *B. arietans* are complex, consisting of a diverse mixture of toxin isoforms from several distinct protein families. Previous transcriptomic and proteomic characterisations show that these venoms consist predominantly of snake venom metalloproteinases (SVMPs), phospholipases A_2_ (PLA_2_), C-type lectin-like proteins (CLPs), disintegrins, snake venom serine proteases (SVSPs), cysteine-rich secretory proteins, L-amino acid oxidases (LAAOs), and kunitz-type serine protease inhibitors [18,19,20,21,22]. However, the venom compositions of these species vary considerably. For example, in Nigeria, the most abundant venom proteins in *B. arietans* venom are CLPs, followed by SVMPs [22], while Nigerian *E. romani* venom is composed mostly of SVMPs, with CLPs, PLA_2_, and serine proteases at lower levels [19,21].

While both *E. romani* and *B. arietans* venoms cause cytotoxicity, the toxins responsible for causing this morbidity remain unclear. Previous studies have shown that members of the SVMP, PLA_2_, and LAAO snake venom toxin families can all exhibit cytotoxic effects [1,23,24]. In addition to their high relative abundance in many viper venoms, SVMPs (which are zinc-dependent) are associated with causing diverse functional activities presenting as local and systemic haemorrhage, coagulopathy, oedema, myonecrosis, blistering, and dermonecrosis in snakebite patients [25,26,27]. SVMPs are structurally classified into three main groups, the PI, PII, and PIII sub-classes, with PI SVMPs containing only a metalloproteinase domain, the PIIs a metalloproteinase and a disintegrin domain, and the PIIIs a metalloproteinase, a disintegrin-like, and a cysteine-rich domain [28]. Each of these subclasses cause distinct pathology in snakebite patients, with PIs considered to be mostly fibrinogenolytic, PIIs predominantly inhibiting platelet aggregation, and PIIIs causing haemorrhage and coagulopathy [23]. However, a previous study by Freitas-de-Sousa et al. [29] showed that dermonecrosis following envenoming by the South American pit viper *Bothrops atrox* was attributed to both PI and PIII SVMPs, which were among the most abundant toxins found in the venom [30]. While there are multiple groups of PLA_2_, only two of these classes are found in snakes: class I in elapids and colubrids, and class II in vipers [31]. Viperid PLA_2_ are known to cause local and systemic myotoxicity, cytotoxicity, and necrosis of skeletal muscle, with numerous studies reporting this from *Bothrops* species [9,32,33,34,35,36,37]. L-amino acid oxidases are known to contribute to venom toxicity and can be cytotoxic and apoptotic, although there is not yet a clear consensus on the exact mechanisms responsible for this [38]. Costal-Oliveira et al. [39] showed LAAO from *B. atrox* caused cytotoxicity in vitro using human epidermal keratinocytes, while LAAO from *Cerastes* venom has been shown to cause necrosis in vivo [40].

As a first step towards target identification of morbidity-causing toxins found in medically important African vipers, in this study, we sought to identify the toxins responsible for causing venom-induced cytotoxicity induced by *B. arietans* and *E. romani*. To do so, we employed a combination of cell- and enzyme-based in vitro assays coupled with chromatographic profiling and separation of the crude snake venoms into their toxin constituents. Our findings demonstrate that SVMP toxins are largely responsible for the cytotoxic effects of both snake venoms observed in cellular assays using human epidermal keratinocytes; however, we found that PIII isoforms are largely responsible for the cytotoxic effects of venom from the saw-scaled viper *E. romani*, while PI SVMPs underpin venom cytotoxicity caused by the puff adder *B. arietans*.

## 2. Results

### 2.1. Saw-Scaled Viper and Puff Adder Venoms Cause Cell Cytotoxicity in Human Epidermal Keratinocytes

**Figure 1:**
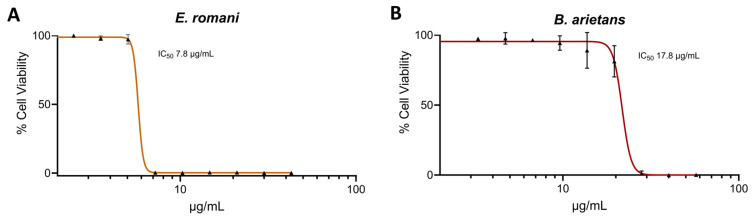
Crude venoms from the saw-scaled viper and puff adder affected cell viability. Cell viability was measured in immortalised human keratinocytes (HaCaT cells) using MTT assays. HaCaT cells were treated for 24 h with serial dilutions of *E. romani* (**A**) and *B. arietans* (**B**) venoms, displayed here as concentration-response curves. The data shown represent mean % cell viability and corresponding standard deviations. All data displayed are from three independent experiments, with each condition conducted in triplicate. The data were normalised to 0–100% between the lowest and highest read values for analysis, then plotted as concentration-response curves using GraphPad Prism 9.

We first investigated the cytotoxic potential of *E. romani* and *B. arietans* venoms in a previously described cell cytotoxicity assay using immortalised human epidermal keratinocytes (HaCaT cell line) [41,42]. The HaCaT cells were exposed to crude venoms at various concentrations for 24 h, followed by the addition of thiazole blue tetrazolium (MTT) assaying to assess cell viability via measures of metabolic activity [43,44]. Both venoms were potently cytotoxic in vitro, with no significant differences seen in the IC_50_ values between *E. romani* venom and *B. arietans* (IC_50_ 7.8 μg/mL ± 2.7 vs. IC_50_ 17.8 μg/mL ± 6.2, respectively) (Figure 1).

### 2.2. Venom Fractions Responsible for Causing Cell Cytotoxicity Are Rich in SVMP Toxins

**Figure 2:**
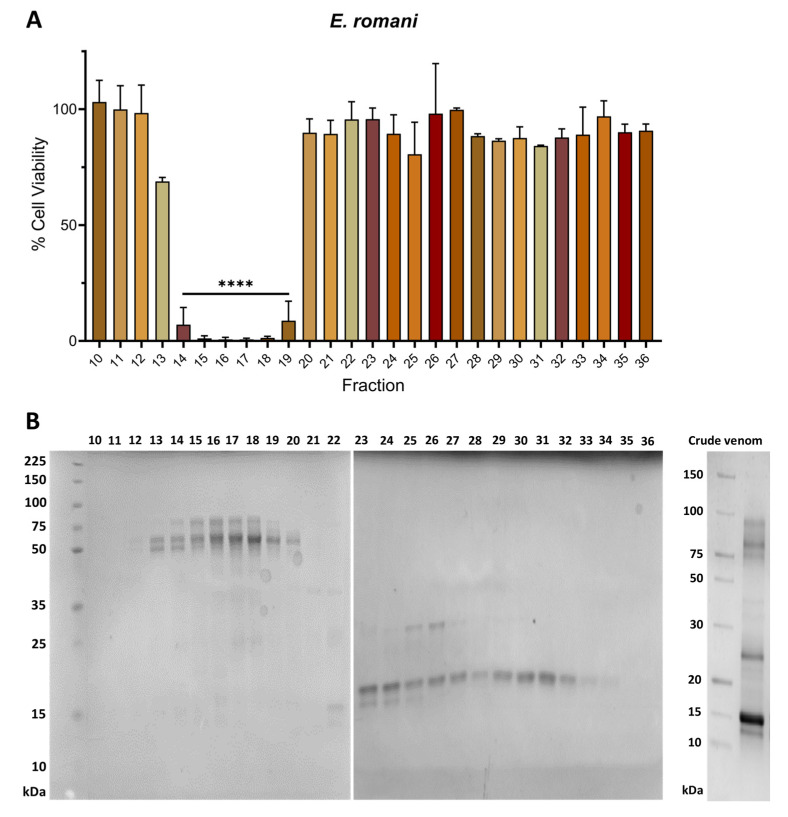
Cytotoxic venom fractions from *E. romani* correlate with the presence of PIII SVMP toxins. (**A**) Cell viability was measured in immortalised human keratinocytes (HaCaT cells) using MTT assays. HaCaT cells were treated with fractions of Nigerian *E. romani* venom, and (**B**) visualised using reducing SDS-PAGE gel electrophoresis on two separate gels. Crude *E. romani* venom (ER) was also visualised for comparative purposes (**B**). In panel (**A**), the data shown represent mean % cell viability and corresponding standard deviations. All data displayed are from three independent experiments, with each condition conducted in triplicate. The data were plotted using GraphPad Prism 9. Statistically significant differences were determined by one-way ANOVA, followed by a Dunnett’s multiple comparisons test, and are denoted by asterisks: **** (*p* < 0.0001).

To investigate which toxins in *E. romani* and *B. arietans* venoms are responsible for causing epidermal cell cytotoxicity, we used a chromatographic approach to separate crude venoms into fractions before retesting these fractions for the effects against the HaCaT cell line via MTT assays. Venoms were separated into fractions by carrying out size exclusion chromatography (SEC) under native conditions. The resulting MTT assays showed that *E. romani* venom contained several adjacent venom fractions (fractions 14–19) that exhibited cytotoxic effects, with significant reductions in cell viability to between 0.6% (±0.9) and 8.8% (±14.5) (*p* ≤ 0.0001) of the vehicle control (Figure 2A). When venom fraction cytotoxic activities were aligned with the chromatographic traces generated from the fractionation process, the active fractions in the MTT assay appeared to correlate with the abundant amounts of PIII SVMPs found in this venom (Figure S1). Further supporting this hypothesis, each fraction was subsequently run on SDS-PAGE to determine the molecular mass of the toxins present (Figure 2B). The cytotoxic active fractions further correlated with the presence of PIII SVMPs, as each was shown to contain multiple protein bands ranging from ~50–80 kDa in size. This analysis also revealed the interesting finding that the *E. romani* PI SVMP visible in fractions 25 and 26 on the gel (~25 kDa) did not exert cytotoxic effects on epidermal keratinocytes.

**Figure 3:**
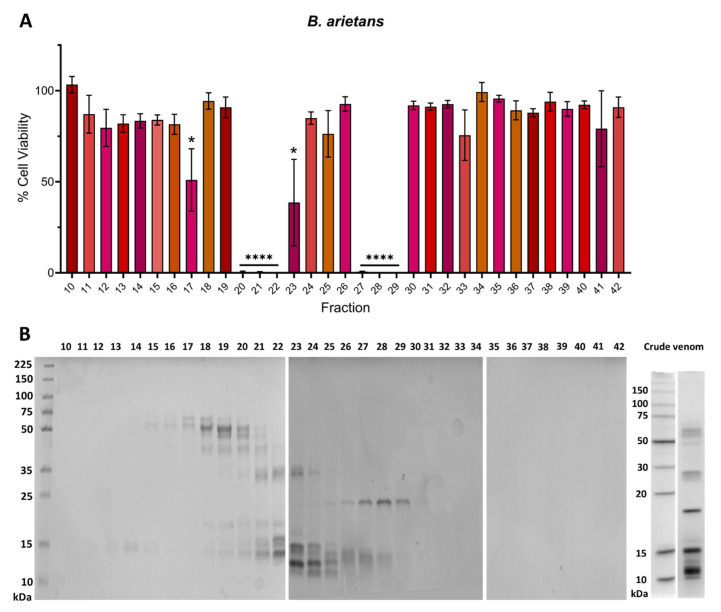
Cytotoxic venom fractions from *B. arietans* correlate with the presence of PII SVMP toxins. (**A**) Cell viability was measured in immortalised human keratinocytes (HaCaT cells) using MTT assays. HaCaT cells were treated with fractions of *B. arietans* venom, and (**B**) visualised using a reducing SDS-PAGE on three separate gels. Crude *B. arietans* venom (BA) was also visualised for comparative purposes (**B**). In panel (**A**), the data shown represent mean % cell viability and corresponding standard deviations. All data displayed are from three independent experiments with each condition conducted in triplicate. The data were plotted using GraphPad Prism 9. Statistically significant differences were determined by one-way ANOVA, followed by a Dunnett’s multiple comparisons test, and are denoted by asterisks: * (*p* < 0.05), **** (*p* < 0.0001).

We used the same approach to identify which *B. arietans* SEC fractions caused cytotoxicity in the MTT assays. As with *E. romani*, multiple SEC fractions were responsible for causing cytotoxicity, with significant reductions in cell viability observed in two main clusters. Cell viabilities were reduced to 0.0% (±0.9) and 0.1% (±1.0) (*p* ≤ 0.0001) for fractions 20–22 and to between 0.0% (±1.1) and 0.6% (±0.4) (*p* ≤ 0.0001) for fractions 27–29 (Figure 3A). When aligned to SDS-PAGE analysis of the fractions, we found protein bands that corresponded with the molecular mass of the recently reported [45] 34 kDa PI SVMP from Nigerian *B. arietans* venom (see fractions 20–23) and the 21 kDa PI SVMP from Tanzanian *B. arietans* venom (see fractions 27–29) (Figure 3B). Additionally, there were multiple bands with masses of ~15–20 kDa in fractions 23–32. We speculate that these may include PLA_2_, disintegrins, or CLP monomers [21], which could potentially contribute to the observed cytotoxicity in fractions 27–29 (Figure 3B). Although there was no cytotoxic activity associated with fractions 18 and 19, which contains serine protease bands at 50–55 kDa [46], we did observe a modest, though still significant, reduction in cell viability in fraction 17 (51.0% ± 29.7; *p* = 0.022), which we hypothesise may contain LAAO, due to this fraction having a band at ~60 kDa (Figure 3A,B).

### 2.3. Metalloproteinase Inhibitor EDTA Significantly Reduces Cytotoxic Effects of Venom Fractions

**Figure 4:**
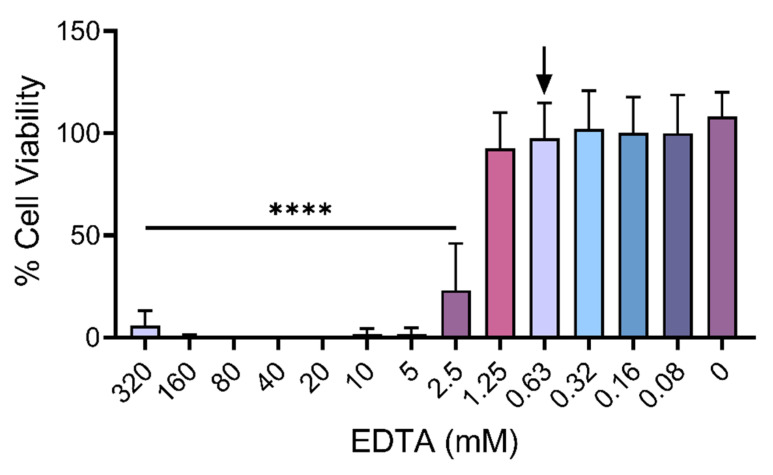
Establishing an appropriate concentration of EDTA to use in cell viability experiments. Cell viability was measured in immortalised human keratinocytes (HaCaT cells) using MTT assays. HaCaT cells were treated for 24 h with serial dilutions of EDTA. The maximal concentration select for use as treatment in subsequent inhibition assays was determined to be 0.625 mM EDTA (black arrow; concentrations displayed in axis are rounded to 2 dp). The data shown represent mean % cell viability and corresponding standard deviations. All data displayed are from three independent experiments, with each condition conducted in triplicate. The data were plotted using GraphPad Prism 9. Statistically significant differences were determined by one-way ANOVA, followed by a Dunnett’s multiple comparisons test, and are denoted by asterisks: **** (*p* < 0.0001).

To further explore the above findings, which suggest that SVMPs play a key role in *B. arietans* and *E. romani* cytotoxic effects, we next performed venom inhibition experiments with the metalloproteinase-inhibiting metal chelator EDTA, which chelates zinc and has previously been used in in vitro neutralisation tests and in vivo experiments investigating protection against venom-induced haemorrhage and dermonecrosis [47,48,49,50]. First, to understand how much EDTA could be used without affecting the viability of the HaCaT cell line, we conducted MTT assays and determined the highest EDTA concentration that could be used for downstream inhibition experiments. We prudently selected the second highest concentration that did not lead to significant reductions in cell viability, which was determined to be 0.625 mM (Figure 4).

**Figure 5:**
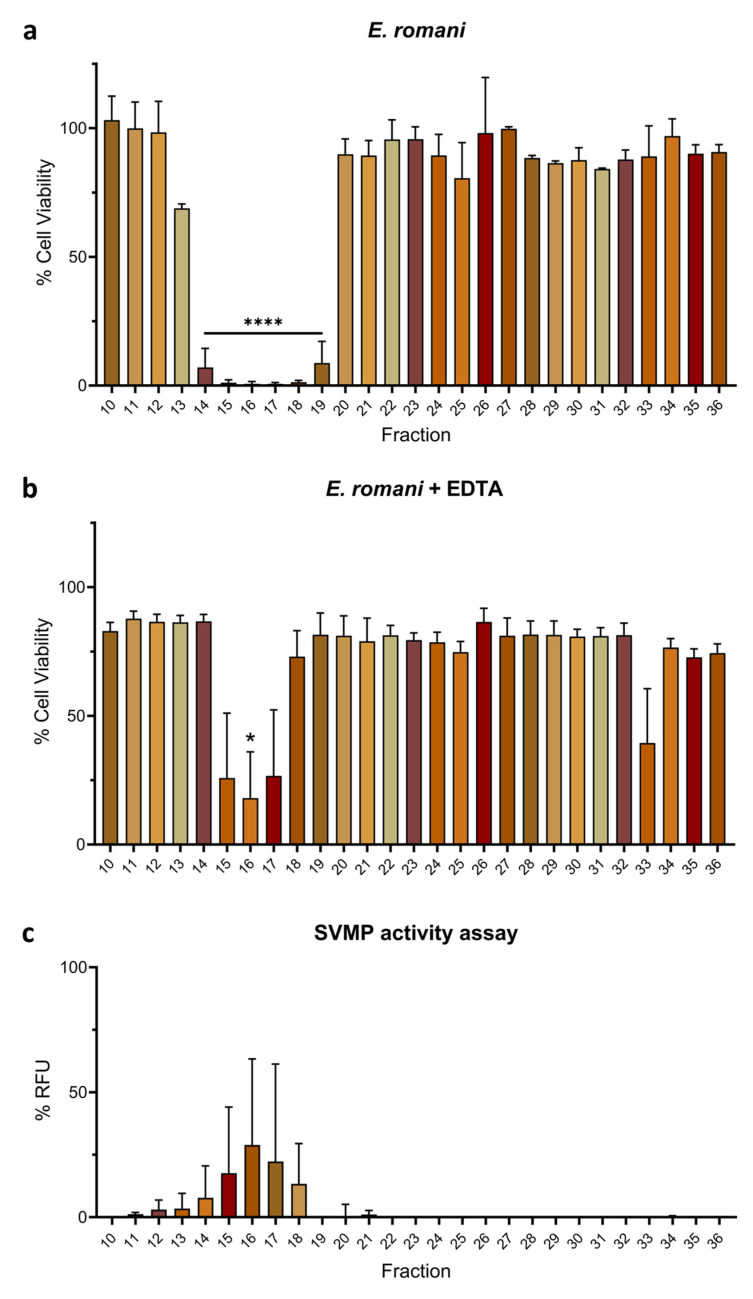
Cytotoxic fractions from *E. romani* venom exhibit SVMP activity and can be inhibited by the SVMP-inhibiting metal chelator EDTA. Cell viability was measured in immortalised human keratinocytes (HaCaT cells) using MTT assays. HaCaT cells were treated with fractions of Nigerian (**a**) *E. romani* venom, and (**b**) *E. romani* venom in the presence of 0.625 mM EDTA. (**c**) A fluorescent-based enzymatic SVMP activity assay showed that venom SVMP activity was observed in the same fractions that were active in the MTT assays (**a**) and were inhibited by EDTA (**b**). In panels (**a**,**b**), the data shown represent mean % cell viability and corresponding standard deviations. The data displayed are from three independent experiments, with each condition conducted in triplicate. In panel (**c**), the data shown represent mean % RFU (intensity measured in relative fluorescence units), relative to crude venom, and corresponding standard deviations. The data displayed are from two independent experiments with each condition conducted in duplicate. The data were plotted using GraphPad Prism 9. In panels (**a**,**b**), statistically significant differences were determined by one-way ANOVA, followed by a Dunnett’s multiple comparisons test, and are denoted by asterisks: * (*p* < 0.05), **** (*p* < 0.0001).

Next, we repeated the earlier MTT assays in the presence of 0.625 mM EDTA to confirm whether cytotoxic activity in the fractions of both venoms was predominately caused by SVMP toxins. Cytotoxicity caused by *E. romani* venom fractions was reduced from six active fractions (fractions 14–19) causing significant reductions in cell viability (*p* ≤ 0.0001) to only one fraction (fraction 16; 18.0% ± 31.1; *p* = 0.040) in the presence of EDTA, with some modest, yet non-significant, cytotoxicity remaining in the adjacent fractions (fractions 15 and 17) (Figure 5a,b). Fraction 16 contained the highest combined content of the ~50–80 kDa proteins, and therefore, remaining activity may have simply been the result of insufficient EDTA to inhibit complete activity of this fraction (Figure 5b). To further explore this relationship between SVMP toxins and venom cytotoxicity, we performed an in vitro fluorescent-based substrate cleavage SVMP enzymatic activity assay [6,51] with the venom fractions. SVMP activity was mostly detected in fractions 15–18 (13.3–28.9% RFU ± 16.2–39.0), with trace activity observed in fractions 11–14 (1.2–7.8% RFU ± 0.6–12.7) (Figure 5c), although variability was high in some fractions. Collectively, these findings demonstrate that cytotoxic fractions from *E. romani* venom contain SVMP toxins and that the cytotoxic activity of these fractions can mostly be inhibited by the SVMP-inhibiting metal chelator EDTA.

**Figure 6:**
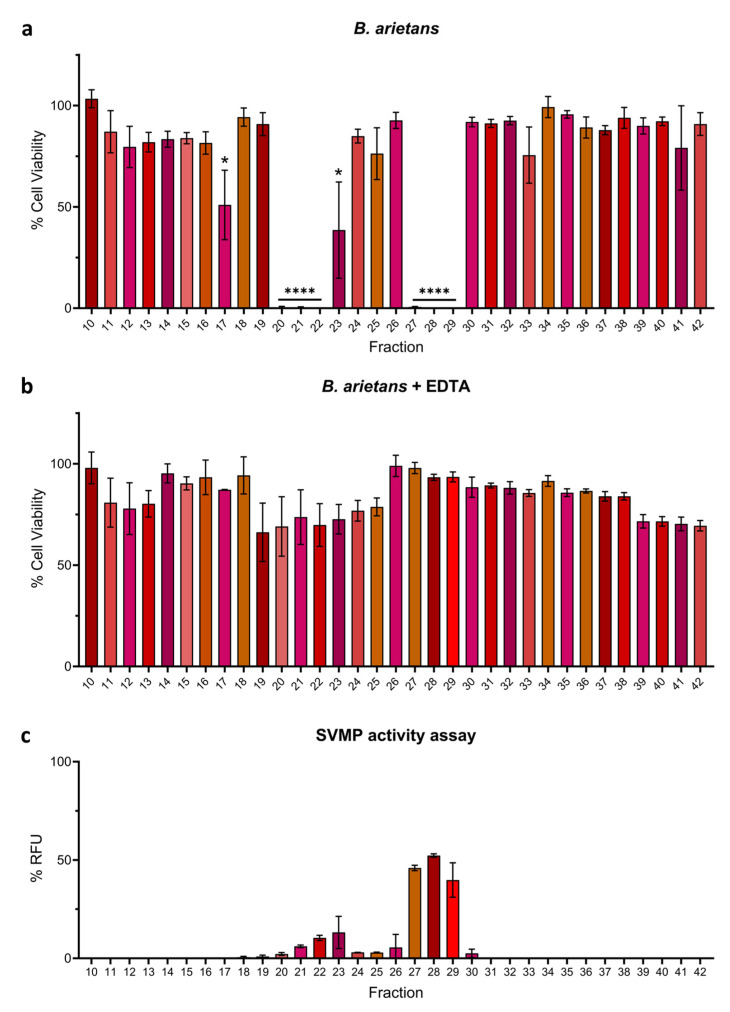
Cytotoxic fractions from *B. arietans* venom mostly exhibit SVMP activity and can be inhibited by the SVMP-inhibiting metal chelator EDTA. Cell viability was measured in immortalised human keratinocytes (HaCaT cells) using MTT assays. HaCaT cells were treated with fractions of (**a**) *B. arietans* venom, and (**b**) *B. arietans* venom in the presence of 0.625 mM EDTA. (**c**) A fluorescent-based enzymatic SVMP activity assay showed that venom SVMP activity was observed in most of the fractions that were active in the MTT assays. In panels (**a**,**b**), the data shown represent mean % cell viability and corresponding standard deviations. The data displayed are from three independent experiments, with each condition conducted in triplicate. In panel (**c**), the data shown represent mean % RFU (intensity measured in relative fluorescence units), relative to crude venom, and corresponding standard deviations. The data displayed are from two independent experiments with each condition conducted in duplicate. The data were plotted using GraphPad Prism 9. In panel (**a**), statistically significant differences were determined by one-way ANOVA, followed by a Dunnett’s multiple comparisons test, and are denoted by asterisks: **** (*p* < 0.0001), * (*p* < 0.05).

EDTA also abolished the activity of cytotoxic venom fractions from *B. arietans.* No reductions in cell viability were observed in the MTT assays for any venom fraction in the presence of 0.625 mM EDTA, which compared favourably to the initial assay where six venom fractions found in two clusters (fractions 20–22 and 27–29) resulted in potent cytotoxic effects (Figure 6a,b). Similar to *E. romani*, findings from the in vitro SVMP activity assay correlated with the MTT assays, with multiple cytotoxic venom fractions displaying SVMP activity, most noticeably fractions 21–23 (6.1–10.4% RFU ± 0.9–1.9) and fractions 27–29 (39.8–52.3% RFU ± 1.2–12.4) (Figure 6c). Low levels of SVMP activity were also observed in fractions 19, 20, 24–26, and 30 (all ≤5.6% RFU), all of which are adjacent to the fractions previously identified as being cytotoxic, though these fractions themselves did not cause significant reductions in cell viability, as measured by the MTT assays (with the exception of fraction 20) (Figure 6). As volumes of fractions used in these experiments were consistent throughout the assay and equal to that used of crude venom (see Supplementary Data Files), the enzymatic activity in this assay totals > 100%, as SVMP-dominated fractions likely contain more activity than in the crude venom at the same comparative volume. Collectively, our results strongly suggest that PI SVMPs largely underpin the cytotoxic effects of *B. arietans* venom observed in human keratinocytes. However, the identity and relative contribution of fraction 17 to venom cytotoxicity remains unclear; though only modestly cytotoxic, the activity of this fraction was effectively inhibited by EDTA, but did not display activity in the SVMP substrate assay (Figure 6).

### 2.4. PI SVMPs from Puff Adders of Differing Geographical Origin Exhibit Substantial Differences in Cell Cytotoxicity

**Figure 7:**
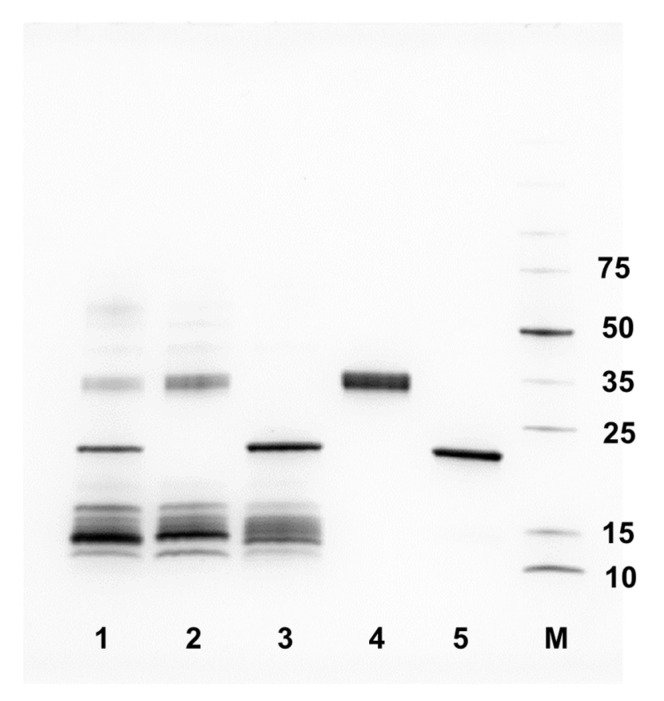
**SDS-PAGE analysis of the *B. arietans* venom samples and purified SVMPs.** The gel was 4–20% acrylamide (BioRad) and was run under reducing conditions. Each lane was loaded with 8 μg of whole venom or 1–2 μg SVMP, and the gel was stained using Coomassie Blue R250. Lane 1, pooled venom; lane 2, Nigerian venom; lane 3, Tanzanian venom; lane 4, purified Nigerian PI SVMP (2 μg); lane 5, purified Tanzanian PI SVMP (1 μg); lane M, markers (Thermo Broad Range) with molecular weights indicated in kDa.

*Bitis arietans* venom is known to vary intraspecifically between different geographical regions (Figure 7) [22,52]. Because we used a pool of venom from Nigerian and Tanzanian specimens for the experiments performed above, and in light of recent data demonstrating distinct substrate specificities of SVMPs sourced from East and West African *Bitis arietans* venoms, we next isolated the different cytotoxic PI SVMPs found in our venom pool by a previously established chromatography approach [45]. Toxin purification showed that the SVMP in fractions 20–22 was characteristic of the recently described Nigerian PI SVMP, while the fractions 27–29 contained the PI characteristic of Tanzanian venom, as demonstrated in a recent publication (see [45]).

**Figure 8:**
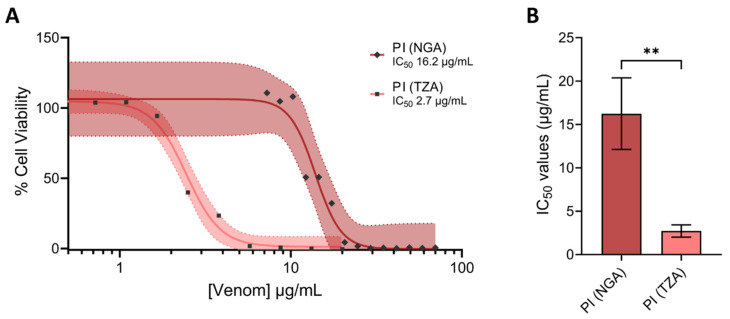
PI SVMPs from puff adder venom exhibited differences in cytotoxic potency. Cell viability was measured in immortalised human keratinocytes (HaCaT cells) using MTT assays. HaCaT cells were treated for 24 h with serial dilutions of PI SVMPs from Nigerian (NGA) and Tanzanian (TZA) *B. arietans* venoms, displayed here as (**A**) 95% confidence bands and (**B**) a summary of the IC_50_ values of the venoms displayed in (**A**). The data shown represent mean % cell viability and corresponding standard deviations. All data displayed are from three independent experiments with each condition conducted in triplicate. In panel (**A**), data were normalised to 0–100% between the lowest and highest read values for analysis, then plotted as 95% confidence band curves using GraphPad Prism 9. In panel (**B**), the data shown represent the mean IC_50_ values of the curves in (**A**) and the corresponding standard deviations. In panel (**B**), statistically significant differences were determined by unpaired t-tests and are denoted by asterisks: ** (*p* < 0.01).

Given the prior description of distinct activities conferred by these two toxins ([45]), we next assessed and compared the relative toxicity of these two isolated proteins against HaCaT cells using MTT assay measures of cytotoxicity. The Tanzanian PI was demonstrated to be significantly more cytotoxic than the Nigerian PI, as demonstrated by the resulting IC_50_ values (IC_50_ 2.7 μg/mL ± 0.7 vs. IC_50_ 16.2 μg/mL ± 4.1; *p* = 0.005) (Figure 8).

### 2.5. Metalloproteinase Inhibitor EDTA Significantly Reduces Cytotoxic Effects of Whole Venoms

**Figure 9:**
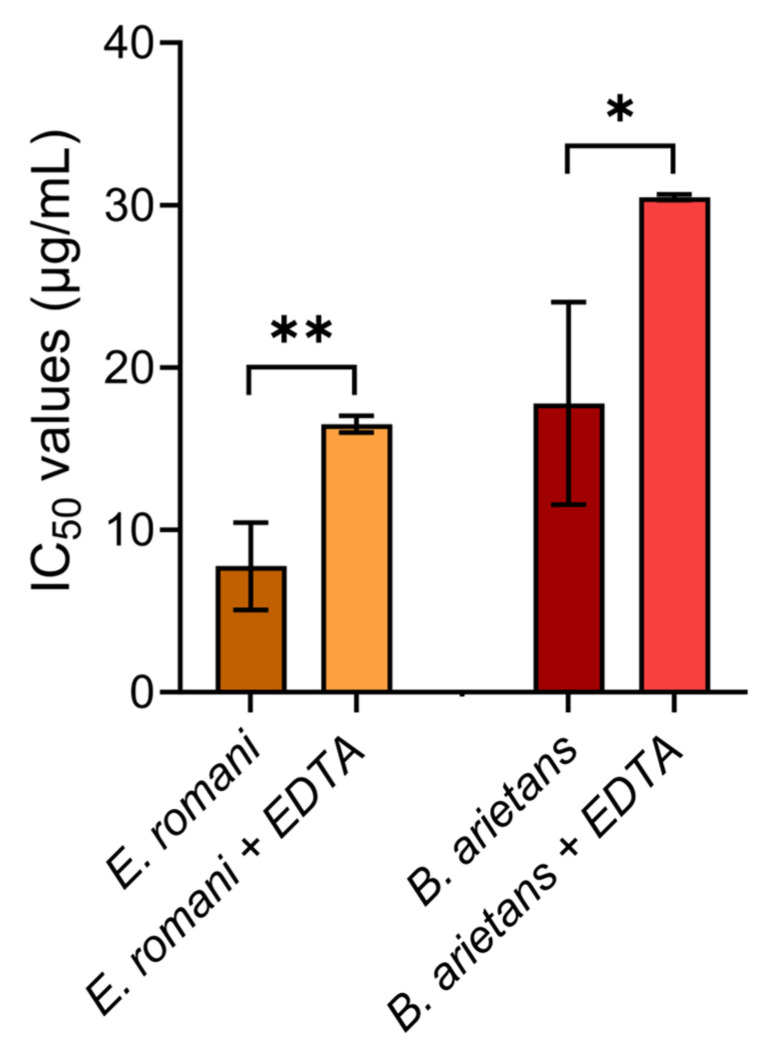
**Cytotoxic crude venom activities are significantly reduced by the metalloproteinase inhibitor EDTA.** Cell viability was measured in immortalised human keratinocytes (HaCaT cells) using MTT assays. HaCaT cells were treated for 24 h with serial dilutions of Nigerian *E. romani* and *B. arietans* venoms, alone and with 0.625 mM EDTA, with the resulting data displayed as IC_50_ values. All data displayed are from three independent experiments, with each condition conducted in triplicate. The data shown represents the mean IC_50_ values and corresponding standard deviations. Statistically significant differences were determined by unpaired t-tests and are denoted by asterisks: ** (*p* < 0.01), * (*p* < 0.05).

As final confirmation that SVMPs are the primary cytotoxic components in vitro, the earlier MTT assays using whole crude venoms sourced from *E. romani* and *B. arietans* were repeated, but this time in the presence of EDTA. HaCaT cells were exposed to serial dilutions of crude venom in the presence of 0.625 mM EDTA, before the cell viabilities were measured as described previously. Cytotoxicity was significantly reduced for both the *E. romani* (IC_50_ 7.8 μg/mL ± 2.7 vs. IC_50_ 16.5 μg/mL ± 0.5; *p* = 0.005) and *B. arietans* (IC_50_ 17.8 μg/mL ± 6.2 vs. IC_50_ 30.5 μg/mL ± 0.2; *p* = 0.024) venoms when incubated with the metalloproteinase-inhibiting metal chelator EDTA (Figure 9).

## 3. Discussion

Snakebites result in upwards of 2.5 million envenomings each year [53] and currently rely on antivenom as the sole therapeutic to treat patients [1]. Due to issues surrounding antivenom safety, efficacy, and affordability [3], work is ongoing to improve antivenom and develop alternative or adjunct treatments, such as monoclonal antibodies and small molecule inhibitors [54]. Such therapeutic modalities differ from mixtures of polyclonal antibodies by targeting specific toxin isoforms, and therefore, it is critical to have a complete understanding of which venom toxins are of greatest pathogenic importance to target for neutralisation. Consequently, in this study, we sought to understand which venom toxins present in saw-scaled viper and puff adder venoms are responsible for causing cytotoxicity, which is associated with contributing to severe local envenoming pathology and causing long-term snakebite morbidity.

Using venoms from *E. romani* and *B. arietans*, we first used cell cytotoxicity assays with human epidermal keratinocytes to demonstrate that both venoms cause cytotoxic effects. This in vitro cytotoxicity has been documented previously, and both venoms are known to be capable of causing local tissue necrosis in human patients [42,55,56]. To identify the toxins responsible for this medically relevant pathology, we used a chromatographic fractionation approach for both venoms, followed by cell cytotoxicity assays and SDS-PAGE gel electrophoresis. In both venoms, the active fractions that were cytotoxic contained SVMPs which we hypothesised may be a key cause of this toxicity, though different molecular masses indicated that PIII SVMPs are likely responsible for cytotoxic effects caused by *E. romani* venom, while those detected in *B. arietans* venom were PI SVMPs [45]. Inhibitory assaying further suggested that SVMPs are the responsible toxins, as the metalloproteinase inhibitor EDTA significantly reduced the cytotoxic effects of the venom fractions and crude venom.

Previous studies using venom from *Bothrops* species have also shown that SVMPs are responsible for cytotoxicity and causing dermonecrosis. Freitas-de-Sousa et al. [29] found both the PI SVMP Atroxlysin-Ia and the PIII Batroxrhagin from *B. atrox* venom led to the rapid onset of dermonecrotic lesions in mice. Furthermore, the small-molecule SVMP inhibitors marimastat (a matrix metalloproteinase inhibitor) and DMPS (a metal chelator) have previously been shown to reduce in vitro cell cytotoxicity caused by *B. asper*, *Daboia russelii*, *E. carinatus*, *E. romani*, and *Crotalus atrox* venoms, thereby hinting at broad SVMP-mediated toxicity [42]. Interestingly, no significant reduction in cytotoxicity was observed when *B. arietans* venom was treated with marimastat and DMPS [42], though this may simply reflect that a higher dose of these drugs is needed or that these inhibitors are less effective against PI toxins compared to PIII SVMPs. Additionally, the use of marimastat and DMPS in vivo have previously shown reductions in haemorrhagic or dermonecrotic dermal lesion sizes following intradermal injection of *C. atrox* or *E. ocellatus* (now reclassified as *E. romani*) venoms in mice [6], suggesting that, for these venoms at least, in vitro cytotoxicity experiments are potentially predictive of in vivo toxicity. Furthermore, identifying the SVMP sub-classes responsible for causing cytotoxicity may allow for more specific targeting of relevant toxins through drug development, with consideration for the structural differences between these sub-classes [23]. Given our demonstration here that SVMPs are responsible for causing cytotoxicity caused by *B. arietans* venom, and that the SVMP-inhibiting metal chelator EDTA significantly reduces these cytotoxic effects, there may be considerable value in dose optimising ‘druggable’ inhibitors, such as marimastat and DMPS [6], to rigorously explore their potential to prevent the local morbidity-causing effects of viper venoms. This is particularly pertinent given that different SVMP toxins were found to be responsible for toxicity in different puff adder samples, with the Tanzanian PI SVMP being significantly more cytotoxic than the PI found in Nigerian venom. While intraspecies venom variation has been previously reported in *Bitis arietans* [22], and also several other venomous snake species [57,58,59,60,61,62], the clinical implications of such variation remains unclear due to a scarcity of case reports describing puff adder envenomings in the literature [63]. It would be valuable to understand whether both these toxins cause severe local envenoming in vivo and whether the differences in cytotoxic potency observed here in vitro result in distinctions in in vivo toxicity and/or the neutralising efficacy of toxin inhibitors or current antivenom therapy.

Although EDTA significantly reduced the cytotoxic effects caused by *E. romani* and *B. arietans* crude venoms, some cytotoxicity remained. While it is possible that residual cytotoxicity may have been reduced further if higher amounts of EDTA were able to be used without causing cell toxicity, there is also the possibility that other less abundant components in the venom may play a role or work in a collective manner (i.e., additive/synergistic) to contribute to venom cytotoxicity, whether that be in combination with the detected cytotoxic SVMPs or other venom components. However, their cytotoxic effects may have been lost when the venom was fractionated into distinct constituents. We recently showed this for the venom of a spitting cobra (*Naja nigricollis*), where PLA_2_ toxins that were weakly cytotoxic by themselves enhanced the cytotoxic activity of other venom components when combined together [41]. The possibility of synergistic or additive effects between toxins within the two viper venoms studied here should, therefore, be investigated in future work, particularly since it is possible that testing venom fractions for cytotoxicity may not completely represent whole venom activity. Recapitulating the fractionated venom minus the SVMP-containing fractions or combining the bioactive fractions with other seemingly inactive fractions would be a robust follow-up study to further explore whether toxin potentiation occurs in *E. romani* and *B. arietans* venoms.

Despite the interesting findings presented in this study, there are several limitations. Firstly, we only investigated in vitro cytotoxicity. Our cell-based assay measures of cytotoxicity used a monolayer of keratinocytes, representing a simplistic model compared with in-development 3D organoid or organotypic models and ex vivo skin models, which may better recapitulate local envenoming in vivo via the combined complexity of multiple skin layers and cell types [41]. Furthermore, we used a metabolic assay for the in vitro cell experiments, which may not always directly correlate to cell viability [64]. Expanding the work performed here to additional cell lines and incorporating more advanced models of cytotoxicity in future studies, such as those outlined above, would be informative in this regard. A further consideration regarding our fractionation work, mentioned above, is that toxicity is often mediated by relative abundance [65,66], and toxins that exist in these venoms at low concentrations will have a reduced impact during bioassaying compared to more abundant toxins. While SVMPs in both venoms were clearly responsible for at least some of the cytotoxicity observed in our cell-based assay, and therefore, it is highly probable that these toxins would substantially contribute to causing dermonecrosis in vivo, other studies have highlighted distinctions between cell-based and in vivo findings for the same reasons as outlined above [41]. Therefore, follow-up preclinical experiments exploring the haemorrhagic and dermonecrotic activity of the isolated SVMP cytotoxins would be valuable to confirm these findings and to contextualise the therapeutic value of SVMP-inhibiting drugs. Finally, the work presented in this study focused on two viperid venoms from Sub-Saharan Africa. Given that venom variation can be extensive across all taxonomic levels [67], and has previously been documented from different geographical locales of *B. arietans* [22,52], it would be valuable to perform similar experiments using venom sourced from specific locales across the broad geographical range of this species. Additionally, it might be informative to extend these studies to include other medically relevant *Echis* or *Bitis* species found throughout the continent.

In summary, our study has shown that SVMPs found in saw-scaled viper and puff adder venoms cause venom cytotoxicity in cellular assays using human keratinocytes. While the toxins in these venoms are different, representing the PI and PIII structural subclasses of SVMP toxins in *B. arietans* and *E. romani* venoms, respectively, both sets of toxins are members of the same protein family and are inhibited by the metal chelator EDTA. The identification of key pathology-causing toxins, such as those described here, is essential for the rational design and development of novel therapeutics that seek to mitigate the severe life- and limb-threatening consequences of tropical snakebites.

## 4. Materials and Methods

### 4.1. Materials

Thiazolyl blue methyltetrazolium bromide (MTT; M5655), NaHCO3 (401676), NaCl (S7653-1KG), and Coomassie brilliant blue G (B-0770) were purchased from Sigma (Gillingham, UK). Dulbecco’s modified Eagle’s medium (DMEM; 11574516), foetal bovine serum (FBS; 11573397), glutaMAX supplement (35050038), penicillin–streptomycin (11528876), phosphate-buffered saline (PBS; 11503387), and TrypLE Express were purchased from Gibco (Loughborough, UK). PageRuler Prestained Protein Ladder 10 to 180 kDa (26616), 2-mercaptoethanol (BP176), tween-20 (11417160), methanol (M/4000/PC17), and glycine (J64365.A1) were purchased from Thermo Scientific (Altrincham, UK). EDTA [N sodium form] (BIA4892) was purchased from Apollo Scientific Ltd. (Stockport, UK). 4–20% Mini-PROTEAN TGX 10-well Precast Protein Gels (4561093) were purchased from BioRad (Watford, UK). Gel-loading dye (purple 6X, B7024S) was purchased from New England Biolabs (Ipswich, UK). Tris base (30-20-60) and SDS (30-33-60) were purchased from Severn Biotech Ltd. (Kidderminster, UK). Acetic acid glacial (20104.334) was purchased from VWR (Lutterworth, UK). SVMP substrate (ES010) was purchased from Bio-Techne (Abingdon, UK).

### 4.2. Snake Venoms

Venoms were sourced from snake specimens maintained in the Liverpool School of Tropical Medicine’s (LSTM) herpetarium. This facility and its snake husbandry protocols are approved and inspected by the UK Home Office and the LSTM Animal Welfare and Ethical Review Boards. Venoms were extracted from wild-caught animals, namely *Bitis arietans* (mixed batch containing venom from both Nigerian and Tanzanian specimens, 13 individuals) and *Echis romani* (Nigeria, 38 individuals in venom pool). Crude venoms were lyophilised and stored at 4 °C to ensure long-term stability. The venoms were resuspended at the desired concentrations in PBS (pH 7.4) prior to experiments.

### 4.3. Venom Toxin Fractionation and Isolation

The protein components of the venoms used in this study were separated using size exclusion chromatography (SEC). For this, 5 mg of venom (*Echis romani* or *Bitis arietans*) was dissolved in 0.5 mL ice-cold PBS (25 mM sodium phosphate, pH 7.2, 0.15 M NaCl) and centrifuged at 10,000× *g* for 10 min. The supernatant was immediately loaded onto a 24 mL column of Superdex 200HR (Cytiva) equilibrated in PBS. The column was operated at a flow rate of 0.2 mL/min during loading and 0.5 mL/min for elution. Fractions of 0.5 mL were collected after the void volume, and elution was monitored at 214 and 280 nm. SDS-PAGE analysis was carried out on all fractions seen to contain protein on the trace. The fractions were stored at −20 °C until use. For work involving purified toxins, Nigerian and Tanzanian PI SVMPs were isolated and identified as described by Wilkinson et al. [45].

### 4.4. Cell Culture

The immortalised human epidermal keratinocyte line, HaCaT [68,69], was purchased from Caltag Medsystems (Buckingham, UK). The cells were cultured in phenol red-containing DMEM with GlutaMAX supplemented with 10% FBS, 100 IU/mL penicillin, 250 µg/mL streptomycin, and 2 mM sodium pyruvate (standard medium; Gibco, Loughborough, UK), per Caltag’s HaCaT protocol. The cells were split, and the growth medium was changed twice weekly up to a maximum of 30 passages. The cells were maintained in a humidified, 95% air/5% CO_2_ atmosphere at 37 °C (standard conditions).

### 4.5. MTT Measures of Cell Metabolic Activity

To determine the cytotoxic effects of crude snake venoms and venom toxin fractions, MTT assays were performed. MTT cell viability [43] assays were completed as described previously [42], with minor modifications. Briefly, HaCaT cells were seeded at 20,000 cells/well in clear-sided/clear-bottomed 96-well plates in standard medium and incubated overnight in standard conditions. The following day, standard medium was aspirated and replaced with treatment solutions prepared in 1% FBS-DMEM medium. Wells were treated in triplicate with 100 μL of crude venoms sourced from *E. romani* (dilution factor 0.49; concentrations: 2.47, 5.04, 10.29, 20.98, and 42.85 μg/mL) or *B. arietans* (dilution factor 0.49; concentrations: 3.29, 6.72, 13.72, 28.00, and 57.14 μg/mL), then placed back in standard conditions for a further 24 h. The treatment solutions were then aspirated and replaced with MTT-containing 1% FBS-DMEM medium (120 μL at 0.83 mg/mL), and the plates incubated for 45 min in standard conditions. Thereafter, the MTT-containing medium was aspirated, 100 μL DMSO was added to each well to dissolve the formazan crystals, and absorbance (550 nm) was read for all wells on a CLARIOstar plate reader (BMG Labtech, Aylesbury, UK). The experiments were repeated independently three times for each venom. Subsequently, the data were normalised to 0–100% between the lowest and highest absorbance values for analysis to represent the %-cell viability (MTT), then plotted as either concentration–response curves or as 95% confidence band curves using GraphPad Prism 9 (v.9.5.1). The IC_50_ values were calculated using the ‘[Inhibitor] vs. normalised response — Variable slope’ — Variable slope’ function.

The above process was repeated using venom fractions from each venom to identify cytotoxins, and then with isolated Nigerian and Tanzanian PI SVMPs to compare the relative cytotoxicity between these two variants. Cells were seeded as described above and treated in triplicate with 100 μL of 1% FBS-DMEM medium containing a neat fraction, at a 9:1 ratio of medium/fraction (or medium/PBS in the case of the control). Subsequent incubation steps, MTT treatments, and measurements were carried out as described above. The experiments were repeated independently three times for each fraction, with the %-cell viability (non-normalised) data plotted as bar graphs using GraphPad Prism 9 (v.9.5.1). Subsequently, purified Nigerian PI (7.26–70.00 µg/mL) or Tanzanian PI (0.72–20.00 µg/mL) SVMPs were tested in the format described above for crude venoms. The data were normalised to 0–100% between the lowest and highest absorbance values for analysis to represent the %-cell viability and plotted as 95% confidence band curves, using the GraphPad Prism 9 (v.9.5.1) function ‘[Inhibitor] vs. response — Variable slope (four parameters)’.

### 4.6. Inhibition of Cytotoxic Effects by EDTA

To assess whether venom SVMPs were responsible for cell cytotoxicity, we performed neutralisation experiments with the metalloproteinase-inhibiting metal chelator EDTA. First, to determine how much EDTA was tolerated by the cell line, HaCaT cells were seeded at 20,000 cells/well in 96-well plates in standard medium, as described above, and incubated overnight under standard conditions. The wells were treated in triplicate with 100 μL of 1% FBS-DMEM with varying concentrations of EDTA (78.1 μM–320.0 mM). After 24 h of incubation under standard conditions, MTT treatments and measurements were carried out as described above. The experiments were repeated independently three times. Subsequently, the data (non-normalised) were plotted as a bar chart using GraphPad Prism 9 (v.9.5.1). From this, the maximal concentration of EDTA used in downstream assays was determined to be 0.625 mM, defined as the second highest concentration that did not significantly reduce cell viability.

Next, we tested whether EDTA could inhibit the SVMP action of both venom fractions and crude venoms. The previously described MTT assays were repeated with 0.625 mM EDTA added to each treatment, with MTT additions and %-cell viability readings carried out as above. The experiments were repeated independently on three occasions. The data for each fraction (non-normalised) were plotted as bar graphs using GraphPad Prism 9 (v.9.5.1). The data for both crude venoms were normalised as above, then plotted as 95% confidence band curves using GraphPad Prism 9 (v.9.5.1). The IC_50_ (MTT) values were calculated using the ‘[Inhibitor] vs. normalised response — Variable slope’ — Variable slope’ function.

### 4.7. SDS-PAGE Gel Electrophoresis

To visualise which proteins were present in each venom, reducing sodium dodecyl sulphate–polyacrylamide gel electrophoresis (SDS-PAGE) was performed using neat venom fractions. Briefly, a 10 μL fraction in 10 μL of reducing SDS-PAGE buffer (6X stock solution with 30% β-mercaptoethanol) was denatured at 100 °C for five minutes. Following this, a 10 μL sample or protein marker (26616, Thermo Scientific, Altrincham, UK) was loaded onto each lane of BioRad Mini-PROTEAN TGX precast (10 well, 4–20%) gels. The gels were run at 200 V for 30 min, before being transferred into Coomassie blue solution (45% methanol, 45% distilled H_2_O, 10% acetic acid glacial, 0.25 g Coomassie blue to 100 mL), and left for one hour at room temperature on a shaker. These were rinsed with destain solution (45% methanol, 45% distilled H_2_O, 10% acetic acid glacial) and left in fresh destain overnight at room temperature on a shaker, before imaging on a GelDoc Go Imaging System (v.3.0.0.07) (BioRad, Watford, UK).

### 4.8. Quantifying Snake Venom Metalloproteinase Activity

To quantify the SVMP activity of the various venom fractions, a previously described SVMP activity assay was performed [6,51]. Fractions of *E. romani* and *B. arietans* venom were used in duplicate, with *E. romani* fractions used at the concentration resulting from chromatographic separation, while *B. arietans* fractions were added at a 1:9 dilution (in PBS, pH 7.4) due to neat venom fractions resulting in signals too high for accurate quantification. Fifteen microlitres of each sample were added to a clear-sided, clear- and flat-bottomed 384-well plate, alongside a DMSO negative control. The ES010 SVMP substrate stock (6.2 mM in DMSO; R&D Systems, Abingdon, UK) was diluted to 9.1 μM in Tris-HCl buffer (150 mM NaCl, 50 mM Tris-HCl; pH 7.5), before 75 µL was added to each well of the plate using a Viaflo 384 (Integra-Biosciences, Thatcham, UK), leading to a final concentration of 7.5 μM once added to the wells. The plate was placed immediately into a CLARIOstar Plus (BMG Labtech, Aylesbury, UK) and read for ten minutes (Ex320 nm, Em420) using CLARIOstar Control software (v6.20). The assay was conducted at room temperature. Generated %-RFU values for each fraction were normalised to the values obtained from crude venom. The data were plotted as bar graphs using GraphPad Prism 9 (v.9.5.1).

### 4.9. Statistical Analysis

All data from cell-based assays are presented as the mean average ± standard deviation of at least three independent experimental replicates. For cell experiments, ‘n’ is defined as an independent experiment completed at a separate time from other ‘n’s within that group of experiments; all drug and/or venom treatments within an ‘n’ were completed in triplicate wells and the mean taken as the final value for that one trial. All data from the SVMP-substrate assays are presented as the mean average ± standard deviation of two independent experimental replicates. Unpaired t-tests were performed for dual comparisons, one-way analysis of variances (ANOVAs) were performed for multiple comparisons, with one independent variable followed by Dunnett’s multiple comparisons tests, as recommended by GraphPad Prism 9 (v.9.5.1). A difference was considered statistically significant where *p* ≤ 0.05.

## Data Availability

The data are available at EMBL-EBI BioStudies (DOI:10.6019/S-BSST1746).

## Supplementary Materials

The following supporting information can be downloaded at https://www.mdpi.com/article/10.3390/toxins17070328/s1: Figure S1: Gel filtration chromatography of whole *E. romani* venom; Figure S2: Gel filtration chromatography of whole *B. arietans* venom.
