# Supplementary material for: Snake Venom Metalloproteinases from Puff Adder and Saw-Scaled Viper Venoms Cause Cytotoxic Effects in Human Keratinocytes
Source: Toxins (Basel). 2025 Jun 28;17(7):328. doi: 10.3390/toxins17070328 (PMC12298971; doi:10.3390/toxins17070328)
Supplement: Supplementary file 1 [file toxins-17-00328-s001.zip › toxins-3402983-supplementary.pdf]

Snake Venom Metalloproteinases from Puff Adder and Saw-Scaled Viper Venoms Cause Cytotoxic Effects in Human Keratinocytes

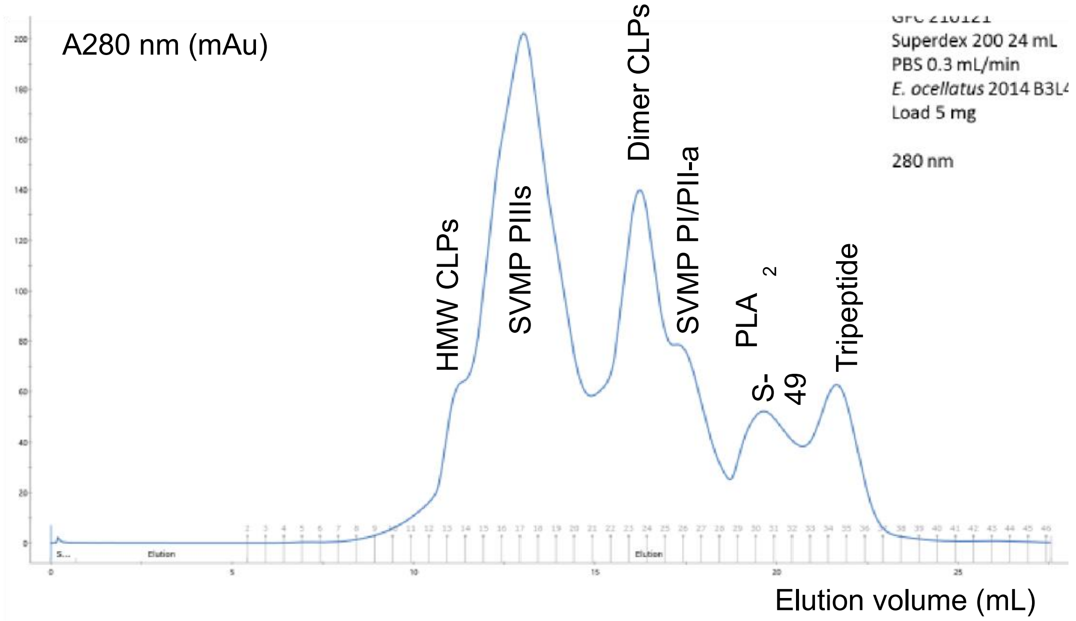

Figure S1. Gel filtration chromatography of whole *E. romani* venom. A 24 mL Superdex 200 column was used for the separation which was pre-equilibrated in PBS [25 mM sodium phosphate, 0.15 M NaCl, pH 7.2]. A 0.5 mL aliquot of *E. romani* venom at a concentration of 10 mg/mL in PBS was injected onto the column and the separation was carried out in PBS at a flow-rate of 0.3 mL/min. Elution was monitored at 280 nm and 0.5 mL fractions were collected. [HMW = high molecular weight].

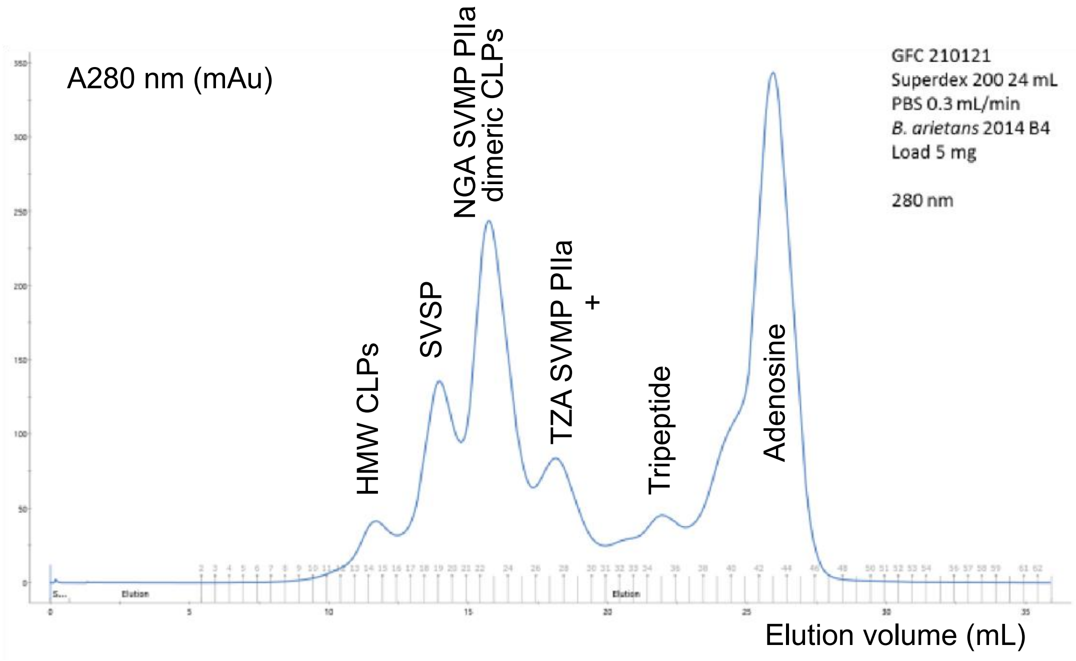

Figure S2. Gel filtration chromatography of whole *B. arietans* venom. A 24 mL Superdex 200 column was used for the separation which was pre-equilibrated in PBS [25 mM sodium phosphate, 0.15 M NaCl, pH 7.2]. A 0.5 mL aliquot of *B. arietans* venom at a concentration of 10 mg/mL in PBS was injected onto the column and the separation was carried out in PBS at a flow-rate of 0.3 mL/min. Elution was monitored at 280 nm and 0.5 mL fractions were collected. [HMW = high molecular weight; CLP = C-type lectin like protein; SVSP = serine protein, NGA = Nigerian; TZA = Tanzanian]
